# The Impact of Unilateral Sensorineural Hearing Loss on Quality of Life of Sub-Himalayan Population

**DOI:** 10.1155/2022/8304639

**Published:** 2022-05-31

**Authors:** Rachit Sood, Saurabh Varshney, Kartikesh Gupta, Nongthombam Surjalata Devi, Narendra Kumar, Amit Kumar Tyagi, Amit Kumar

**Affiliations:** ^1^Department of ENT, AIIMS, Rishikesh, Uttarakhand, India; ^2^Department of ENT, AIIMS, Deogarh, Jharkhand, India; ^3^Department of ENT, Shyam Shah Medical College, Rewa, Madhya Pradesh, India

## Abstract

**Introduction:**

Unilateral Sensorineural Hearing Loss (USNHL) is an age-old known entity. Patients with USNHL lack the merits of binaural hearing, i.e., temporal summation, sound localization, and speech recognition in a noisy environment. The lack of binaural hearing affects such individuals' quality of life (QOL). The present study is the most extensive Indian series related to QOL in USNHL patients.

**Objectives:**

This study aims to assess the impact of USNHL on the QOL of patients in the state of Uttarakhand.

**Methods:**

A cross-sectional study was carried out at a tertiary care center in Uttarakhand over a period of 18 months, from January 2018 to July 2019. A total of 115 patients with pure USNHL were evaluated using a validated questionnaire—Hearing Handicap Inventory for Adults-Hindi (HHIA-H).

**Results:**

In our study, patients with higher HHIA-H scores were male, young adults (age group 18–30 years), students, and laborers. Most of our patients (64%) had significant handicaps, followed by moderate handicaps in 25%. However, no significant correlation of degree of handicap was seen with age, duration, and degree of hearing loss.

**Conclusion:**

USNHL can lead to a significant handicap that can severely affect the emotional and social aspects of life. Thus, early diagnosis and rehabilitation are essential to prevent handicap and uplift QOL in patients with USNHL. This trial is registered with Clinical Trials Registry of India (CTRI reg. no. CTRI/2018/06/014396).

## 1. Introduction

Hearing is one of the five essential senses of our body that helps us communicate with one another and helps gain awareness of one's surroundings. According to World Health Organization (WHO), a person who is not able to hear as well as someone with normal hearing, i.e., a hearing threshold of 20 decibels (dB) or better in both ears, is said to have hearing loss. This was revised in 2020 over the previous threshold of 25 dB. Hearing loss has emerged as a global health challenge that at present has affected more than 466 million people, around 5% of the world's population [1]. The number of hearing loss patients in India is above 63 million, more than 6% of the population [2]. Unilateral sensorineural hearing loss (USNHL) has been defined as hearing loss in one ear with an air-bone gap of less than 15 decibels, with the other ear being normal. The incidence of USNHL varies from 3.2–19.4%. [3]. Patients with USNHL lack the advantages of binaural hearing, which includes temporal summation, sound localization, and most importantly, the ability to understand speech in a noisy environment [4]. USNHL can be caused by various cause such as idiopathic sudden SNHL, trauma, acoustic neuroma, Meniere's disease, mumps, infections, noise induced, and common variable immunodeficiency [5].

Over decades, the definition of health has changed from the mere absence of disease to multifaceted entities comprising of physical, social, and mental well-being. Overall well-being can be assessed by a patient's quality of life (QOL), which is defined by WHO as “individual's perception of their position in life in the context of culture and value systems in which they live and in relation to their goals, expectations, standards, and concerns” [6]. An innumerable number of questionnaires have been developed to assess the QOL of a patient.

Very limited epidemiological data exists in the literature till date on the prevalence and effect of USNHL in India and QOL. Hence, this particular study aims to assess the impact of USNHL on QOL of patients in India using a previously validated questionnaire, Hearing Handicap Inventory for Adults-Hindi (HHIA-H) [7]. The present study is the most extensive series of QOL in USNHL patients in India and the first to assess in the most commonly spoken language, i.e., Hindi.

## 2. Methods

An observational cross-sectional study was conducted in the Department of Otorhinolaryngology in a tertiary care hospital in Uttarakhand, India, over a period of 18 months from January 2018 to June 2019. After clearance from the Institutional Ethical Committee (IEC/18/102), the study was registered with the Clinical Trials Registry of India (CTRI/2018/06/014396). All patients visiting the outpatient department fitting the inclusion criteria: (a) age between 18 and 50 years and (b) USNHL (other ear being normal) based on pure tone audiometry (PTA) were enrolled. Patients with comorbidities, conductive or mixed hearing loss in either ear, and any history of ear discharge from either ear were excluded. A detailed history was taken, and examination was performed after taking written informed consent. The patients were then asked to fill HHIA-H. WHO grading of hearing loss was used. Audiometric thresholds were measured at 500, 1000, 2000, and 4000 Hz. The hearing loss was classified as mild (20 to 34.9 dB), moderate (35 to 49.9 dB), moderately severe (50 to 64.5 dB), severe (65 to 79.9 dB), profound (80 to 94.5 dB), and complete deafness (95 dB and above) [8].

HHIA-H questionnaire: the questionnaire comprised of a total of 25 items with scores for responses, which were “Yes” (4 points), “Sometimes” (2 points), and “No” (0 points). The total score ranged from 0 to a maximum of 100. 12 items represented the social subscale (maximum score = 48) and 13 items represented the emotional subscale (maximum score = 52). Handicap was graded as “Nil” (score 0 to 17), “Mild to moderate” (score 18 to 43), and “Significant” (score of 44 and above).

Data analysis: the responses of USNHL patients to HHIA-H were documented using Microsoft Excel. Statistical analysis was performed using SPSS Statistics for Windows, version 21.0 (IBM Corp., Armonk, NY).

## 3. Results

115 patients were studied with a male preponderance of 71%. The majority of patients were aged between 18 and 30 years (50%), with a mean of 31.7 years. 30.4% of the patients who presented with USNHL were students. There was a slight preponderance of left side laterality (54.8%), and the majority were suffering from profound hearing loss of >80 dB (54.8%), with an average hearing loss of 83.46 dB. The duration of hearing loss in most patients ranged from 1 to 5 years (36.5%), with an average of 39.38 months (Table 1).

The overall HHIA-H mean score was 52.21 with a standard deviation of 25.20 and a median score of 56. The social subscale had a mean of 26.21 with a standard deviation of 12.65 and a median of 26. The emotional subscale had a mean of 25.97 with a standard deviation of 13.82 and a median of 26.

The majority of the study population was found to be suffering from a significant handicap (63.5%), followed by mild to moderate handicap (25.2%) and nil handicap (11.3%).

**Figure 1:**
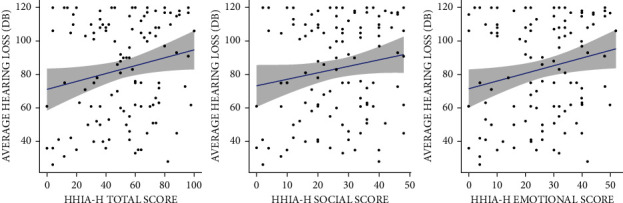
Scatter plot showing correlation between HHIAH scores, subscores, and average hearing loss.

HHIA-H scores were correlated with age, average hearing loss, and duration of hearing loss. Nonparametric tests (Spearman correlation coefficient-rho) were used, as at least one of the variables was not normally distributed. These correlations have been depicted using a scatter plot. A statistically insignificant weak positive correlation was seen between HHIA-H total score and average hearing loss (rho = 0.18, *p*=0.052). A similar statistically insignificant, weak positive correlation was seen between HHIA-H social score and average hearing loss (rho = 0.13, *p*=0.151). However, a statistically significant weak positive correlation was found between HHIA-H Emotional Subscale and the average hearing loss (rho = 0.20, *p*=0.0366). For every 1 dB increase in average hearing loss, the HHIA-H score increased by 2.17 units (Figure 1). There was no statistically significant correlation seen between overall HHIA-H scores with age (rho = −0.11, *p*=0.257) and duration of hearing loss (rho = −0.12, *p*=0.218). The HHIA-H scores were normally distributed in both ears, and hence, parametric tests (*t*-test) were used to make group comparisons. However, no significant difference was seen in HHIA-H scores between the ears affected (*t* = 0.993, *p*=0.323).

## 4. Discussion

Understanding of USNHL has grown over time from being ignored to being acknowledged by both patients and medical professionals. The only study in the state of Uttarakhand on the epidemiology of USNHL was conducted by Tyagi et al. [3] of which 155 patients (8.6%) were found to be suffering from pure USNHL, which is comparable to our sample size of 115. HHIA was initially developed in the English language by Newman et al. in 1990 [9]. Since then, it has been translated and validated into various languages such as Italian [10], Malay [11], Kannada [12], and Hindi [7] (HHIA-H). Many studies have used multiple questionnaires to assess the QOL in bilateral hearing loss, but minimal literature exists on the impact on QOL in USNHL patients.

Most patients were young adults in our study, similar to Augustine et al. [13]. Maximum HHIA-H score was seen in the young population (age group 18–30 years), comparable to the age group 31–40 years. This can be attributed to the working male population and occupation-directed questions in HHIA-H. In our study, patients with profound hearing loss had higher HHIA-H scores because they had maximum difficulty in hearing, which was accordant with the other study [13]. In our study, patients with prolonged duration of illness had a minimal score, discordant with the other study [13]. It might be due to the adaptation of disability over time.

In our study, there was no correlation found between HHIA-H scores and age, gender, side of the ear affected, or duration of hearing loss, which was comparable with the findings of Augustine et al. [13].

HHIA handicap was compared to previous similar studies (Table 2). In our study, 89% of the population suffered from handicap. Only 11% of the population reported no handicap, which is accordant with Newman et al. [14] and Araújo et al. [15]. However, our results were not comparable with the study conducted by Augustine et al. [13]. This difference in outcome can be attributed to Augustine et al. having used the English version of HHIA in the non-English speaking population. Since HHIA is a self-report questionnaire, it should ideally be in the language that the subject can easily understand to assess the handicap accurately, as in our study.

A weak positive correlation was seen between HHIA-H total, emotional, and social subscale scores with average hearing loss in our study. However, this correlation was statistically insignificant with the total score and social subscale but significant with the emotional subscale. Hence, individuals with a higher hearing loss would face more emotional handicaps. The HHIA-H score of the emotional subscale increased by 2.17 units for every 1 dB increase in average hearing loss. However, no statistically significant correlation with the total score was consistent with Newman et al. [14].

Our study had similar HHIA-H scores for the right and left ear in USNHL, and no significant association was seen between HHIA-H scores and the laterality of USNHL. This was similar to the results of Augustine et al. [13]. The right ear has a physiological advantage in speech processing because the left cerebral hemisphere is the chief site for speech procession for the majority of the population, and 75% of the cochlear nerve fibers cross to the opposite side. Thus, the speech coming to the right ear reaches the left hemisphere directly, where the speech presented to the left ear reaches the right hemisphere and then crosses over to the left hemisphere for processing. Hence, HHIA-H scores should be higher in the right ear USNHL, as described by Jerger et al. in 1995 [16].

“Disabling hearing loss” refers to patients with hearing loss of more than 40 dB in the better hearing ear in adults (15 years or older) [17]. However, it was noticed in our study that the degree of handicap was not correlated to the degree of hearing loss, which makes us rethink that patients with mild hearing loss might also be suffering from a significant handicap, as seen in our study. This may under define the actual burden of disabled patients.

The auditory input from both ears sums up to show a synergistic effect to amplify the sound almost two times when it reaches the auditory cortex. This summation is absent in USNHL. The interaural time and intensity difference helps a person to localize the sound source. Hence, USNHL patients suffer from impaired sound localization, and this can have an adverse impact on daily routine activities such as crossing a road safely. The binaural squelch effect enables a person to differentiate speech from noise due to the head shadow effect. USNHL patients lack this advantage, and this can cause a significant impact on the patients, both socially as well as emotionally. The disadvantages of lack of binaural hearing can be dealt with hearing rehabilitation options such as hearing aids and bone-anchored hearing aid [18].

To better understand and analyze QOL in multiple aspects of a patient's life with USNHL, we need further studies with a larger sample size to establish such significant correlations.

## 5. Conclusion

USNHL can have varying impacts on the QOL of patients. The lack of binaural hearing impairs sound localization and understanding speech in a noisy environment. This can profoundly impact all aspects of an individual's life. Significant handicap is seen in young adult males and individuals from low socioeconomic strata. HHIA is a self-report questionnaire. Hence, it needs to be in the native language of the study population. It is essential to diagnose these patients, grade their handicaps, and rehabilitate them appropriately.

## Figures and Tables

**Table 1:** HHIA profile of patients with USNHL (*N* = 115).

HHIA-H demography *(N*=115)					
—	—	Frequency (*N*=115)	Percentage	Mean HHIA-H	Standard deviation
Gender	Male	82	71	52.22	25.275
	Female	33	29	52.18	25.418

Age	18–30	57	50	54.95	22.779
	31–40	30	26	52.13	25.167
	41–50	28	24	46.71	29.641

Education	Upto high school	64	56	54.34	25.629
	Graduates	35	30	54.80	22.451
	Postgraduates	16	14	38.00	26.005
Occupation	Laborer	25	22	57.60	24.440
	Student	35	30	55.09	21.830
	Teacher	10	09	41.80	31.488
	Businessman	18	16	47.11	29.243
	Others	27	23	50.74	24.646

Income	<Rs 20000	66	57	52.73	25.153
	Rs 20001–40000	37	32	54.27	23.646
	>Rs 40001	12	11	43.00	30.109

Ear	Right	52	45	54.81	26.801
	Left	63	55	50.06	23.808

Hearing loss	Mild	4	03	29.50	03.109
	Moderate	15	13	39.60	03.906
	Moderately severe	20	18	57.35	05.122
	Severe	13	11	73.00	03.439
	Profound	10	09	88.40	03.921
	Complete deafness	53	46	111.43	29.757

Duration	<1 month	18	16	53.00	27.079
	1 month-1 year	37	32	53.24	28.132
	1–5 years	42	36	55.76	23.665
	>5 years	18	16	41.00	18.243

HHIA-H handicap	Nil	13	11	08.15	
	Mild-moderate	29	25	32.62	
	Significant	73	64	67.84	

**Table 2:** HHIA handicap comparison among different studies.

HHIA-H handicap	HHIA Newman et al. (1997) [14] (*N*=43)	HHIA-P Araujo et al. (2010) [15] (*N*=52)	HHIA Augustine et al. (2013) [13] (*N*=50)	HHIA-H present study (2019) (*N*=115)
Nil	28%	27%	54%	11%
Mild-moderate	32%	48%	31%	25%
Significant	40%	25%	15%	64%

## Data Availability

The “Master Chart Excel File” data used to support the findings of this study article.

## Additional Points

The identity and details of each of the participants were kept confidential and were not disclosed.
